# Relocation of *Sr48* to Chromosome 2D Using an Alternative Mapping Population and Development of a Closely Linked Marker Using Diverse Molecular Technologies

**DOI:** 10.3390/plants12081601

**Published:** 2023-04-10

**Authors:** Vallence Nsabiyera, Naeela Qureshi, Jianbo Li, Mandeep Randhawa, Peng Zhang, Kerrie Forrest, Urmil Bansal, Harbans Bariana

**Affiliations:** 1 School of Life and Environmental Sciences, Faculty of Science, The University of Sydney Plant Breeding Institute, 107 Cobbitty Road, Cobbitty, NSW 2570, Australia; 2 Nabuin Zonal Agricultural Research and Development Institute, National Agricultural Research Organization, Moroto P.O. Box 132, Uganda; 3 International Maize and Wheat Improvement Center (CIMMYT), Carretera Mexico-Veracruz Km. 45, El Batan, Texcoco C.P. 56237, Mexico; 4 International Maize and Wheat Improvement Center (CIMMYT), World Agroforestry Centre (ICRAF Campus), UN Avenue, Gigiri, Nairobi P.O. Box 1041-00621, Kenya; 5 Agriculture Victoria, AgriBio, Centre for AgriBioscience, 5 Ring Rd., Bundoora, VIC 3083, Australia; 6 School of Science, Faculty of Science, Hawkesbury Campus, Western Sydney University, Richmond, NSW 2753, Australia

**Keywords:** stem rust, *Sr48*, translocation, FISH, GISH, marker-assisted selection (MAS), Ug99, wheat

## Abstract

The Ug99-effective stem rust resistance gene *Sr48* was mapped to chromosome 2A based on its repulsion linkage with *Yr1* in an Arina/Forno recombinant inbred line (RIL) population. Attempts to identify markers closely linked to *Sr48* using available genomic resources were futile. This study used an Arina/Cezanne F_5:7_ RIL population to identify markers closely linked with *Sr48*. Using the Arina/Cezanne DArTseq map, *Sr48* was mapped on the short arm of chromosome 2D and it co-segregated with 12 markers. These DArTseq marker sequences were used for BlastN search to identify corresponding wheat chromosome survey sequence (CSS) contigs, and PCR-based markers were developed. Two simple sequence repeat (SSR) markers, *sun590* and *sun592*, and two Kompetitive Allele-Specific PCR (KASP) markers were derived from the contig 2DS_5324961 that mapped distal to *Sr48*. Molecular cytogenetic analysis using sequential fluorescent in situ hybridization (FISH) and genomic in situ hybridization (GISH) identified a terminal translocation of chromosome 2A in chromosome 2DL of Forno. This translocation would have led to the formation of a quadrivalent involving chromosomes 2A and 2D in the Arina/Forno population, which would have exhibited pseudo-linkage between *Sr48* and *Yr1* in chromosome 2AL. Polymorphism of the closet marker *sunKASP_239* among a set of 178 wheat genotypes suggested that this marker can be used for marker-assisted selection of *Sr48*.

## 1. Introduction

Common wheat (*Triticum aestivum* L., 2n = 6x = 42, AABBDD) is among the world’s most widely grown crops, with an annual acreage of 220 million ha [1]. Its acreage is ranked second, next to rice, in developing countries. Its global trade volume surpasses all other major food crops combined [2]. It provides about one fifth of the recommended daily calories, and its contribution to protein is greater than that of maize [1].

Stem rust, also known as black rust, caused by *Puccinia graminis* f. sp. *tritici* (Pgt), has historically been a devastating disease of common wheat and durum wheat in Africa, Asia, Australia, Europe, the Middle East and New Zealand [3]. Yield losses of 20–30% from stem rust were reported during the mid-20th century in many geographical regions, such as eastern and central Europe [4], Asia, Australia, Europe and the United States [3]. The favorable environment for disease development and cultivation of susceptible cultivars has the potential to lead to destruction of the whole crop within three weeks of disease onset [3,5].

The detection of Pgt pathotype Ug99 (TTKSK) in Uganda in 1998 [6] rendered a large proportion (90%) of global wheat cultivars susceptible [7]. Pathotype TTKSK rapidly migrated to neighboring wheat-growing countries, including Tanzania, Kenya, Ethiopia and Sudan, and has subsequently been detected in the Middle East and South Africa [8]. This pathotype acquired virulence for stem rust resistance genes *Sr24* and *Sr36* in Kenya in 2006 and 2007, respectively [9,10]. In 2013–2014, the Pgt pathotype TKTTF overcame the resistance gene *SrTmp* and caused an epidemic that destroyed 10,000 ha of the widely cultivated wheat variety Digalu in Ethiopia [11].

Various control measures have been used to manage stem rust epidemics, including the removal of the alternate host (*Berberis vulgaris* L.) of Pgt, the use of host plant resistance [7] and the use of fungicides [12]. More than 60 stem rust resistance genes have been formally named in wheat (https://shigen.nig.ac.jp/wheat/komugi/genes/symbolClassList.jsp accessed on 20 September 2022). The deployment of rust resistance genes to control stem rust was estimated to save the Australian wheat industry AUD 12 million annually [13]. The deployment of resistance genes singly has resulted in boom-and-bust cycles through the emergence of new pathogen variants [14]. Watson and Singh [15] emphasized the deployment of combinations of more than one gene following the breakdown of singly deployed genes *Sr6* (Eureka) and *Sr11* (Gabo) in Australia. Although it is possible for a pathogen to acquire virulence for more than one gene at once, the probability of such an event is very low. A successful breeding program for disease resistance relies on the continuous supply of genetically diverse sources of resistance [16,17]. Therefore, identification and characterization of new sources of resistance are crucial to achieve durable disease control.

The Swiss hexaploid winter wheat cultivar Arina was observed to carry an uncharacterized seedling stem rust resistance gene based on multi-pathotype tests by Pathan and Park [18]. Bansal et al. [19] mapped this resistance on the long arm of chromosome 2A based on its repulsion linkage (16.5 cM) with *Yr1* using an Arina/Forno recombinant inbred line (RIL) population, and formally named it *Sr48*. Attempts to identify markers closely linked with *Sr48* using several genomic resources in the distal region of chromosome 2AL were futile. In the current study, we used an Arina/Cezanne RIL population to identify and validate markers closely linked with *Sr48* for use in marker-assisted selection (MAS).

## 2. Results

### 2.1. Rust Response Assessments

**Figure 1:**
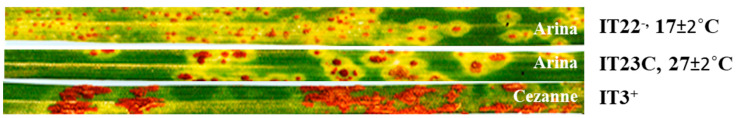
Infection types (ITs) produced by Arina (*Sr48*) and Cezanne against pathotype Pgt 98-1,2,3,5,6,7 at 17 ± 2 °C and 27 ± 2 °C.

The Arina cultivar displayed infection type (IT) 22^−^ at 17 ± 2 °C and IT 23C at 27 ± 2 °C, while the susceptible parent Cezanne displayed IT 3^+^ at both temperature regimes against the Pgt pathotype 98-1,2,3,5,6,7 (Figure 1). The Arina/Cezanne RIL population was evaluated at 17 ± 2 °C in three replications, and it showed monogenic segregation (84 RILs of ITs 22^-^ to 22^+^ and 88 RILs of ITs 33^+^ to 3^+^; χ^2^_1:1_ = 0.29, *p* > 0.50 at 1 *d.f*.) for seedling stem rust response.

### 2.2. Construction of DArTSeq Linkage Map

DArTSeq markers were filtered and markers with allele-calling ≥ 98% were selected. SNP markers with a high proportion of missing data (>10%) and rare SNPs with <5% minor allele frequency were discarded. The most informative SNPs were selected based on a threshold PIC value ≥ 0.2. Extra steps; such as (i) imputation of missing data, (ii) genotype correction using the R tool ‘ABHgenotypeR’, (iii) removal of co-located SNPs and (iv) repeated marker ordering were employed for genetic map construction. Finally, 7115 DArTseq markers were used for linkage map construction.

A genetic linkage map was generated using the ASMap package in the R program at a LOD value of 6. The Arina/Cezanne linkage map had 54 linkage groups. The A, B and D genomes had 1429.0, 1298.6 and 925.9 cM map length, respectively (Supplementary Table S1). The D genome was the least saturated of the three genomes. The Arina/Cezanne linkage map covered a genetic distance of 3653.5 cM, with a density of at least 1 marker per 0.52 cM.

### 2.3. Mapping of Sr48

**Figure 2:**
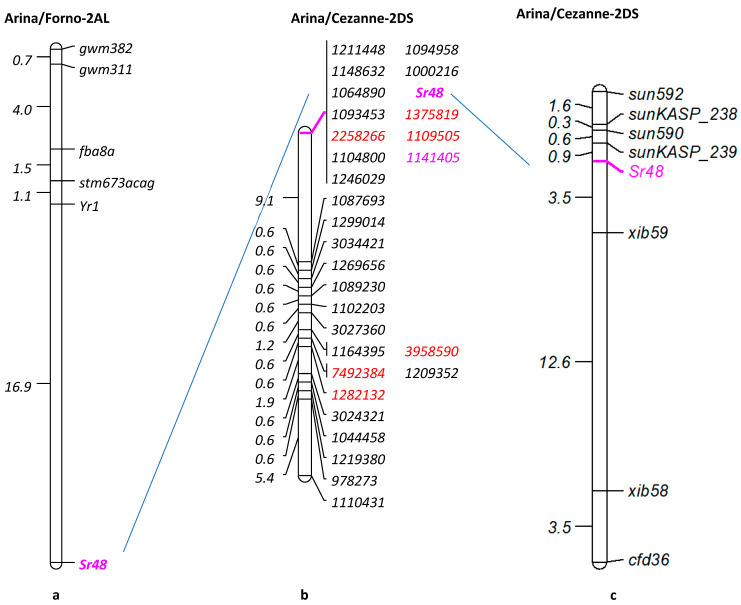
Genetic linkage map showing the location of *Sr48* [19] in chromosome 2AL in Arina/Forno (**a**), the DArTSeq marker map of 2DS on 92 Arina/Cezanne RILs (**b**) and PCR-based marker 2DS map of 172 Arina/Cezanne RILs (**c**).

Seedling IT data for the Arina/Cezanne RIL population was converted into genotypes. The resistant phenotype was converted to ‘A’ and the susceptible to ‘B’, and they were then incorporated into the DArTSeq-based linkage map. The stem rust resistance gene *Sr48* showed co-segregation with 12 markers located on the short arm of chromosome 2D (Figure 2b). A BlastN search of the DArTseq marker sequences linked with *Sr48* identified four CSS contigs (Table 1) that were used to develop PCR-based STS and SSR markers. We were unable to obtain a good hit (100% similarity) for markers *1211448*, *1094958*, *1148632*, *1000216*, *1064890*, *1093453*, *1104800* and *1246029* when BlastN searched for CSS contigs. Eighteen markers were developed and tested for polymorphism on the parents. Two markers, ‘*sun590′* and ‘*sun592′,* from the CSS contig 2DS_5324961 were polymorphic between the parents. Markers *xib59*, *xib58* (published polymorphic markers from chromosome 2DS), *sun590* and *sun592* were genotyped on the entire RIL population (172 lines). The *sun590* marker was co-dominant and amplified 161 bp and 157 bp fragments in Arina and Cezanne, respectively. The *sun592* marker was dominant and amplified a 159 bp amplicon in Arina in addition to a 153 bp amplicon common to both parents. Sanger sequencing of amplicons with STS markers *sun846*, *sun847*, *sun848* and *sun849* (Table 1) from parental lines was performed. Six SNPs were identified when *sun849* parental sequences were compared. These SNPs were converted to KASP assays and named *sunKASP_238* to *sunKASP_243* (Table 2). Primer sequences of these KASP markers are given in Table 2. A genetic map was constructed using polymorphic SSR, STS and KASP markers. Consequently, the *Sr48* map (Figure 2c) included seven markers with *sunKASP*_*239,* which located 0.9 cM distal to *Sr48,* and *xib59* was placed 3.5 cM proximal to *Sr48* (Figure 2c). *Sr48* flanking markers (*sunKASP*_*239* and *xib59*) were also tested on the Arina/Forno RIL population (200 lines). The *sunKASP_239* marker mapped 16.7 cM from *Sr48* and *xib59* segregated independently.

### 2.4. Comparison of Sr48-Linked Markers Developed with Chromosome Survey Sequences with Arina LrFor (Arina) Pseudomolecule and CS Reference Genome Sequence v1.0

**Figure 3:**
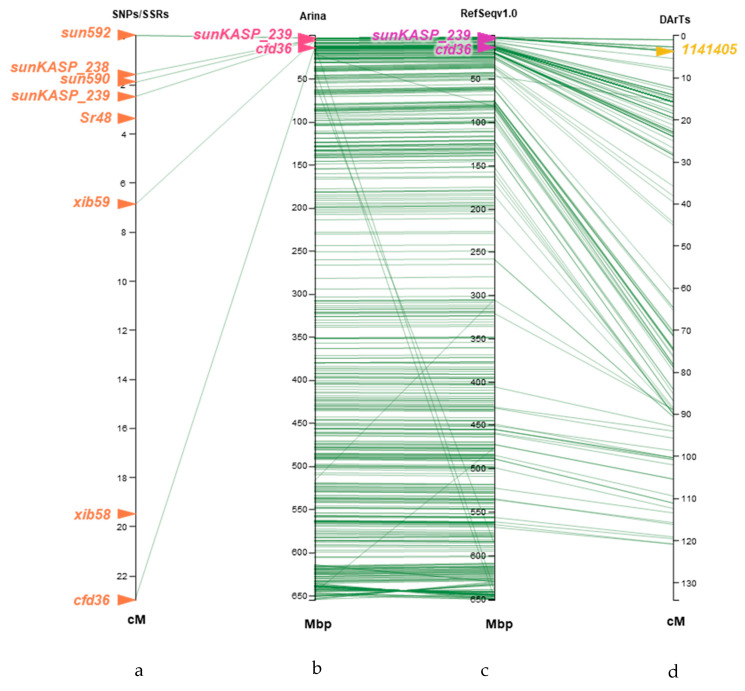
Comparison of location of *Sr48*-linked markers on chromosome 2DS (**a**) Arina 2D pseudomolecule (**b**) IWGSC RefSeqv 1.0-based 2DS (**c**) and DArT markers (**d**) of Arina/Cezanne RIL population using the Pretzel software (https://plantinformatics.io/, accessed on 20 September 2022).

Pretzel software (https://plantinformatics.io/, accessed on 20 September 2022) was used to compare the position of *Sr48* linked markers: SNP/SSR markers (3a), Arina pseudomolecule (3b), Chinese Spring Refseqv1.0 (3c) and DArTs (3d). We observed collinearity, suggesting that the ordering in the genetic map was accurate (Figure 3).

### 2.5. Molecular Cytogenetic Analysis

**Figure 4:**
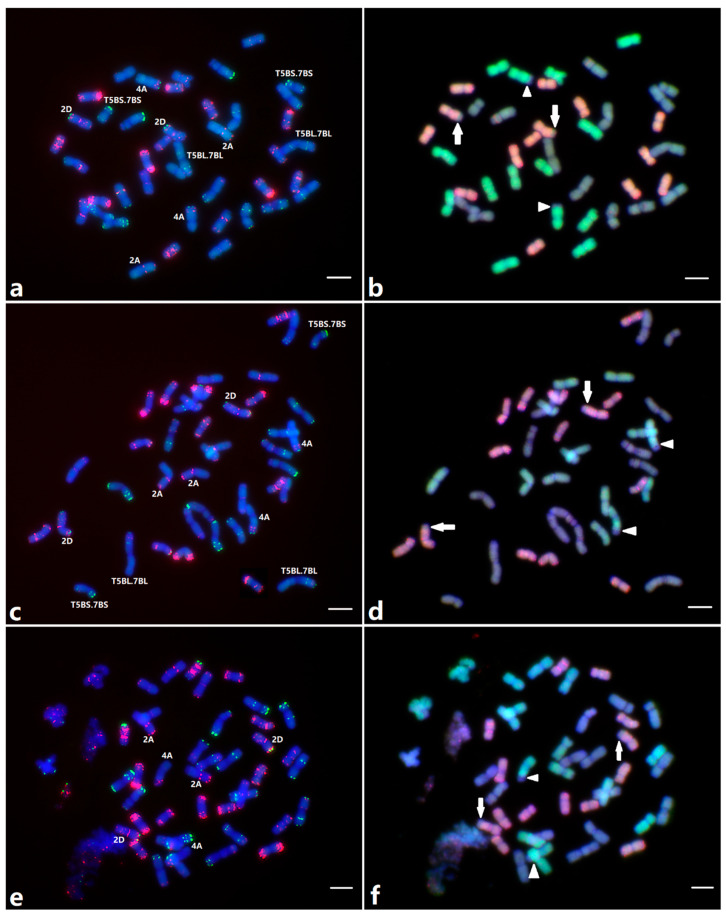
Sequential FISH (**a**,**c**,**e**) and GISH (**b**,**d**,**f**) analyses of cultivars Arina (**a**,**b**), Cezanne (**c**,**d**) and Forno (**e**,**f**). These experiments were performed on the same metaphase cell for each cultivar. For FISH, chromosomes were hybridized with Oligo-pSc119.2-1 probes labeled with 6-FAM and Oligo-pTa535-1 labeled with Tamra, generating green and red signals, respectively. Chromosomes were counterstained with DAPI and fluoresced blue dye. For GISH, DNA from *Tritcum urartu* and *Aegilops tauschii* were labeled with fluorescein-12-dUTP and rhodamine-5-dUTP to differentially fluoresced A and D chromosomes, respectively. Chromosomes were again counterstained with DAPI and fluoresced blue. Arrowheads point to the translocation breakpoints on the 4AL-5AL-7BS translocation chromosomes in all three cultivars. The reciprocal translocation chromosomes T5BS.7BS and T5BL.7BL in Arina (**b**) and Cezanne (**d**) are labeled. Arrows point to the breakpoints on 2DL (interstitial translocations in Arina (**b**) and Cezanne (**d**); distal translocation in Forno (**f**)). Scale bars represent 10 µm.

Sequential FISH and GISH were performed on the cultivars Arina, Forno and Cezanne in order to study whether there were any translocations between chromosomes. The *4AL*-5AL-7BS cyclic translocation found in all hexaploid wheats [20,21] was also observed in all three cultivars (Figure 4b,d,f; arrowhead points to the translocation breakpoint). In addition, both Arina and Cezanne carried a pair of reciprocal translocations, T5BS.7BS and T5BL.7BL (Figure 4a,c) [22]. The GISH results also indicated that there was an interstitial translocation in chromosome 2DL in both Arina and Cezanne (Figure 4b,d); on the contrary, there was a terminal translocation in chromosome 2DL in Forno, most likely from the A genome (Figure 4f). This translocation in Forno might have played a role in the location of *Sr48* on 2AL in the Arina/Forno RIL population.

### 2.6. Polymorphism of the Sr48 Linked Marker sunKASP_239

The closest marker, *sunKASP_239,* for *Sr48* was tested on a set of Australian and European wheat genotypes to determine the polymorphism for its use in the marker-assisted selection. One Australian cultivar (Naparoo) and eight European cultivars (Avle, Blanka, Boru, Dacke, Kadett, Vitus, William and WW 20299) amplified the Arina allele (A:A), and the remaining 169 cultivars amplified the Cezanne allele (G:G). These results indicated 94.9% polymorphism among the Australian and European wheat cultivars, respectively.

## 3. Discussion

Rust diseases, including stem rust, have historically caused severe yield losses in wheat [23,24]. The identification and use of stem rust resistance genes has been an important strategy to effectively control this disease since the 1950s [7,25]. *Sr48* is one of the race-specific resistance genes among over 63 formally designated resistance genes to date [26] https://shigen.nig.ac.jp/wheat/komugi/genes/symbolClassList.jsp, accessed on 20 September 2022) that is effective against Ug99 (H.S. Bariana, unpublished), and can, therefore, be useful when combined with other stem rust resistance genes. Markers linked with this gene can facilitate its rapid selection and pyramiding with other marker-tagged stem rust resistance genes. Bansal et al. [19] located *Sr48* on the long arm of chromosome 2A based on its repulsion linkage with the stripe rust resistance gene *Yr1,* which was previously located on chromosome 2AL [27]. Several unsuccessful attempts were made to find closely linked markers in the Arina/Forno RIL population using various genomic resources.

Repetitive testing of the RIL population at low and high temperatures demonstrated that *Sr48* expresses better at lower temperatures (17 °C) compared to the optimum stem rust incubation temperature of above 25 °C (Figure 1; Table 3). This resulted in robust phenotyping of the Arina/Cezanne RIL population. Temperature sensitivity of stem rust resistance genes, including *Sr6* [28,29], *Sr21* [30], *Sr38* [27] and *Sr52* [31], has been reported in wheat.

The Arina/Cezanne RIL population was originally produced to Mendelize the QTL (*Qsr.sun*-5BL) that was permanently named *Sr56* Bansal et al., 2014 and to determine genomic locations of leaf rust and stripe rust resistance genes present in both parents. *Sr56* is an adult plant stem rust resistance gene; it is located in the long arm of chromosome 5B and it is ineffective at the seedling stage. Therefore, no interference was observed while phenotyping the population for *Sr48*. A set of 92 RILs was genotyped using DArTseq markers, and this information was used for the purpose of developing markers closely linked with *Sr48*. To our surprise, linkage analysis mapped *Sr48* to the short arm of chromosome 2D, contrary to the previous report about the location on the long arm of 2A in the Arina/Forno RIL population [19]. The PCR-based markers (*sun590* and *sun592*) developed from the CSS contig 2DS_5324961, showing homology with the DArTSeq marker 1141405, also confirmed the location of *Sr48* on 2DS in the Arina/Cezanne RIL population. Further, SNP-based KASP markers were developed by sequencing the parental amplicon produced by STS markers. This observation prompted us to genotype Arina and Cezanne parental cultivars using the previously reported markers on chromosome 2DS [32,33,34] and Cao et al., 2012. This resulted in the construction of a chromosome 2DS linkage map that included *sun592*, *sunKASP_238*, *sun590*, *sunKASP_239*, *Sr48*, *xib59*, *xib58* and *cfd36* markers (Figure 2c). Flanking markers *sunKASP_239* and *xib59*, when tested on the Arina/Forno population, did not show the expected linkage, authenticating our previous results.

In order to confirm the discrepancy in the location of *Sr48* in the Arina/Forno and Arina/Cezanne populations, we performed sequential FISH and GISH analyses on the parental cultivars. The objective behind this cytological study was to find out if there was any translocation that could explain the pseudo-linkage between *Yr1* and *Sr48*. Interestingly, GISH results identified a translocation of what was most likely chromosome 2A at the distal end of the long arm of chromosome 2D of cultivar Forno (Figure 4f; arrow points to the breakpoint). We concluded that this translocation might produce quadrivalents to suggest a pseudo-linkage between *Yr1* and *Sr48,* as was observed by Bansal et al. [19].

The relocation of *Sr48* to the short arm of chromosome 2D prompted us to prove the uniqueness of this gene. It is important to examine the relationship of *Sr48* and other stem rust resistance loci reported in this region. Two other stem rust resistance genes, *Sr6* (Tsilo et al., 2009, 2010) and *Sr46,* which originated from diploid wheat Aus18913 (Arora et al., 2019), have been mapped to chromosome 2DS (7.9 Mb of the physical map). *Sr6* expresses seedling IT 0- [29,34], contrary to the intermediate IT 22^−^ displayed by *Sr48* at low temperatures. In addition, *Sr6* is linked with marker *wmc453*, which was mapped at the 69 cM position [33]. The most distal marker *cfd36* in this study was mapped at the 7 cM position [33], which is 62 cM distal to *wmc453.* Therefore, *Sr6* and *Sr48* represent different loci.

**Figure 5:**
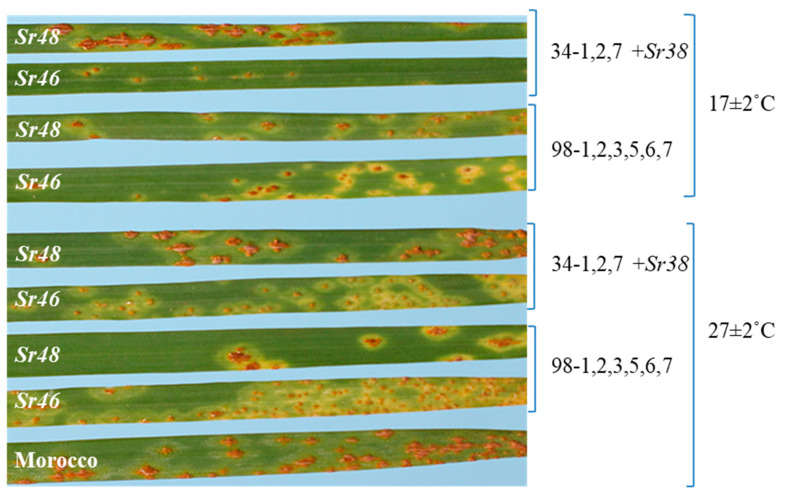
Seedling responses of genotypes carrying *Sr48* (Arina), *Sr46* (hexaploid derivative of Aus18913 progeny number 7.1a) and Morocco (the susceptible check) against Pgt pathotypes 34-1,2,7+Sr38 and 98-1,2,3,5,6,7 when tested at two temperature regimes in the greenhouse.

*Sr46* was first identified from *Ae. tauschii* accession Aus18913 and was cloned by Arora et al. (2019). It exhibits IT 1^+^ to 2^−^ under optimal conditions. We tested Arina (*Sr48*) and a resistant derivative of Aus18913 (donor source of *Sr46*) 7.1a (kindly provided by Dr. Evan Lagudah) against Pgt 34-1,2,7+Sr38, which is virulent on *Sr48* at both low and high temperatures (Figure 5). The line carrying *Sr46* exhibited resistance against this pathotype. *Sr46* was placed at 7.9 Mb of the physical map of Chinese Spring (RefSeq v1.0), whereas the marker *sunKAP_239* was located close to *Sr48* at 2.69 Mb. This comparison indicated that DArTseq marker 1141405 varied from 2.3 to 6.3 Mb in different genomes, but the *sunKASP_239* was placed at 3.88 Mb position in Arina 2D pseudomolecules (Figure 3a–d). The comparison of infection type, phenotypic specificity and genomic locations of *Sr46* and *Sr48* led us to conclude that these genes are different. All these results demonstrated the location of *Sr48* on the short arm of chromosome 2D.

Breeder-friendly KASP marker was developed in this study. The marker *sunKASP_239* mapped 0.9 cM distal to *Sr48* and showed a high level of polymorphism (>94%) in diverse genetic backgrounds. An Australian wheat panel was tested against the different Pgt pathotypes under the Australian Cereal Rust Program, genes were postulated and information was provided to breeders (Bariana per comm). Based on multipathotype tests, it was determined that none of the lines carried *Sr48*. The marker *sunKASP_239* showed the *Sr48*-specific allele in winter wheat cultivar Naparoo, which also carried *Sr24*. Pathotypes that were virulent on *Sr24* are not available in Australia; therefore, the phenotypic data were not sufficient to confirm the presence of *Sr48* in Naparoo. The European wheat panel was tested with five stem rust isolates and the presence of *Sr7b*, *Sr8a*, *Sr12*, *Sr15*, *Sr17*, *Sr23* and *Sr30* was predicted. Out of nine lines that possessed the *Sr48*-specific allele, Blanka, Boru and Kadett carried *Sr15*, whereas Avle carried *Sr15*+*Sr17*. The cultivars Dacke and Vitus were susceptible. Therefore, we concluded that *sunKASP_239* showed false positive amplification in these lines.

Due to its high level of polymorphism, the *sunKASP_239* marker will be useful in marker-assisted selection of *Sr48* and its pyramiding with other marker-tagged genes. Markers for many stem rust resistance genes have been developed (https://maswheat.ucdavis.edu/protocols/stem_rust_protocols, accessed on 20 September 2022), and these also include adult plant resistance genes *Sr2*, *Sr55*, *Sr56*, *Sr57, Sr58* and *Sr63*. The deployment of combinations of all stage resistance and adult plant resistance genes in new wheat cultivars through MAS can ensure durable stem rust control [17].

In conclusion, the development of a new population (Arina/Cezanne) helped us in relocating *Sr48* from the long arm of chromosome 2A to the short arm of chromosome 2D. The presence of a chromosome 2D/2A translocation in Forno was reported for the first time, and it was the cause of the initial location of *Sr48* on the long arm of chromosome 2A based on the pseudo-linkage with *Yr1*. The marker *sunKASP_239* is very closely linked with *Sr48* and thus it can be used for MAS in breeding. The *Sr48*-linked SNP can also be useful in genomic selection-based wheat improvement.

## 4. Materials and Methods

### 4.1. Host Materials

A Swiss winter wheat cultivar, Arina (Zenith/Moisson), was crossed with stem rust-susceptible French winter wheat cultivar Cezanne (Thesee/87B29). F_2_ seeds derived from a single F_1_ plant were harvested and advanced to develop an F_5:7_ RIL population of 172 lines using the single seed descent method. A panel of 178 wheat cultivars were used to test the polymorphism of the marker(s) linked with *Sr48*.

### 4.2. Greenhouse Screening

Parental lines and the Arina/Cezanne RIL population were raised in 9 cm diameter pots filled with potting mix consisting of pine bark and coarse sand (2:1 ratio). The stem rust-susceptible cultivar Morocco was included as a control. The material was fertilized at sowing and six days after germination with Aquasol^®^ at a rate of 20 g/10 L of water. Inoculations were performed according to Bariana and McIntosh (27) with the Pgt pathotype 98-1,2,3,5,6,7 (PBI culture no. 580). Inoculated seedlings were incubated in the greenhouse at 18–20 °C for 48 h in water-filled steel trays covered with hoods made from translucent polythene sheets to allow natural light to enter and to maintain 100% relative humidity. After incubation, infected seedlings were transferred to two microclimate growth rooms maintained at low (17 ± 2 °C) and high (27 ± 2 °C) temperature regimes, respectively, to identify the ideal temperature for better expression of *Sr48*. All Arina/Cezanne RILs were tested three times at a low temperature (17 ± 2 °C) and two times at a high temperature (27 ± 2 °C) with pathotype 98-1,2,3,5,6,7 (using 8–10 seeds of each RIL). Rust response assessments were performed 14–16 days post-inoculation using a 0 to 4 infection type (IT) scale [35].

### 4.3. Genotyping of Arina/Cezzane RIL Population

Genomic DNA was extracted from eight seedlings from each RIL and parental line following the procedure described in Bansal et al. [36]. The quality and quantity of DNA were measured on a 1% agarose gel and using a NanoDrop-ND1000 spectrophotometer (Nanodrop Technologies), respectively. The DNA samples of 92 RILs and parents were sent to Diversity Arrays Technology Pty Ltd., Canberra, Australia for genotyping by sequencing (GBS) with the wheat DArTseq^®^ platform (http://www.diversityarrays.com, accessed on 20 September 2022).

### 4.4. Development of PCR Based Markers

The DArTseq marker sequences (about 40 bp) showing linkage with *Sr48* were used to identify IWGSC Chromosome Survey Sequencing (CSS) using the BlastN search at the INRA server (https://urgi.versailles.inra.fr/blast/?dbgroup=wheat_all&program=blastn, accessed on 20 September 2022). The CSS contigs were searched for repeats using the simple sequence repeat (SSR) identification tool (http://archive.gramene.org/db/markers/ssrtool, accessed on 20 September 2022). The sequence-tagged-site (STS) and simple sequence repeat (SSR) primers were designed using Primer 3 software (http://bioinfo.ut.ee/primer3-0.4.0/primer3, accessed on 6 September 2022). The forward primers were labeled with the M13 sequence CACGACGTTGTAAAACGAC. Polymorphic markers were genotyped on the entire RIL population according to the procedure described by Bansal et al. [37]. In addition to the markers mentioned above, the chromosome 2DS-specific markers *barc124*, *barc95*, *gwm455*, *gwm296*, *gdm5*, *xib59*, *xib58*, *xib3*, *xib66*, *xib87*, *xib100*, *xib89*, *xib128* and *xib90,* reported by Cao et al. [38], and *gwm210*, *cfd36* and *wmc111* from Yu et al. [39], were also used.

The STS amplification products of *Sr48* flanking markers were Sanger sequenced at the Australian Genome Research Facility (www.agrf.org.au, accessed on 20 September 2022) and compared for nucleotide variation using Sequencher software (www.genecodes.com, accessed on 20 September 2022). The SNPs identified in STS sequences were used to design Kompetitive Allele-Specific PCR (KASP) markers using PolyMarker (http://www.polymarker.info/, accessed on 20 September 2022). The STS and KASP markers were named using the prefix *sun* (Sydney University) followed by the consecutive numbers.

### 4.5. Molecular Cytogenetic Analysis

Parental cultivars Arina, Forno and Cezanne were characterized by sequential fluorescence in situ hybridization (FISH) and genomic in situ hybridization (GISH) for chromosomal rearrangements, following the procedures of Li et al. [40] and Zhang et al. [41]. OligopSc119.2-1 and Oligo-pTa535-1 were labeled with 6-carboxyfluorescein (6-FAM) and 6-carboxytetramethylrhodamine (Tamra), producing green and red signals, respectively, and were used for the identification of individual chromosomes. Chromosomes were counterstained with 4′,6-diamidino-2-phenylindole (DAPI) (Invitrogen Life Science, Carlsbad, CA, USA) and fluoresced blue. Slides were analyzed with a Zeiss Axio Imager epifluorescence microscope. Images were captured with a Retiga EXi CCD (charge-coupled device) camera (QImaging, Surrey, BC, Canada) operated with Image-Pro Plus version 7.0 software (Media Cybernetics Inc., Bethesda, MD, USA) and processed with Photoshop version CS6 software (Adobe Systems, San Jose, CA, USA).

After stripping off the oligo probes, the same slides were used in GISH. The total genomic DNA from *T. urartu* (AA genome) and *Aegilops tauschii* (DD genome) was labeled with Fluorescein-12-dUTP and Rhodamine-5-dUTP (Roche Diagnostic Australia, Castle Hill, NSW, Australia), respectively, using nick translation. Unlabeled total genomic DNA of *Ae. speltoides* (SS genome) was used as a blocker. The probe to blocker ratio was ~1:10. Chromosomes were counterstained with DAPI and pseudo-colored blue.

### 4.6. Statistical Analysis

Chi-squared (χ^2^) test of goodness-of-fit was used to compare the observed stem rust response segregation against the expected genetic ratio for a single gene. A linkage map was constructed using the ASMap function in R software [42]. Genetic distances were determined using the Kosambi mapping function [43] to transform recombination frequencies into centiMorgans (cM).The 2D genetic linkage maps of the chromosomes were generated in MapChart [44].

## Figures and Tables

**Table 1:** Primer sequences of STS and SSR markers derived from the wheat chromosome survey sequences (CSS) in the region spanning *Sr48.*

Dartseq Marker	CSS	Position in Physical Map (bp)		STS/SSR (Repeat)	Primer	Forward ^a^	Reverse
		Start Site	Stop Site				
1109505	2DS_6421726	2,944,768	2,944,836	STS	*sun582*	AGTACAGGTCGTGGCTGCTT	ATGTCGCCAAGTTCGCATAG
				STS	*sun583*	GGCAAAATGGCAGGATATTG	TCCATCCATGTTCCTGATGA
2258266	2DS_4785040	2,944,771	2,944,703	STS	*sun584*	AAGGCTGACCTTTGACCTGA	CCGACCTCATACCGGTACAT
1141405	2DS_5324961	2,341,675	2,341,622	STS	*sun846*	ATTACTGCGCCTCCTTCTGA	CGACCATTAGCTGACTGCAA
				STS	*sun847*	GCGGCTTGACATCTCATCTT	CACGTATTCGCAGCAAAAGA
				STS	*sun848*	GTTTTGGGTACCGAGCAAAA	TGCCTCGTTGATTGAAATTG
				STS	*sun849*	CCGTTGTCTCCTCTGAAAGC	GCGTTGTAGGTGCTTTGCTT
				SSR (GCG)3	*sun585*	GTGAATCGTACAAGTGGAATG	ACTCTGCATCGTAAGTCACC
				SSR (CCG)3	*sun586*	TTAAGGCTAAAACAAATCAGG	CGATACAAAGGCAGATGG
				SSR (AGA)3	*sun587*	AAGTAGCATTTCTGGAACACA	AATACTTGCACATCTCCTTTG
				SSR (AGAGA)5	*sun588*	GCTTGCTTCCAGTTCCAG	CTCAAGCAAGCAAATAGTCAT
				SSR (TCGCCG)6	*sun589*	CCGTCCCACAGGTGTCTC	TCTGAACCAAACACATCCTAC
				SSR (TCCT)4	*sun590*	TACTCTAGTGACCAACCAACA	GGCCATTACATGGTACTAGTTT
				SSR (AGCT)4	*sun591*	TTATTCCATTTCAATCTGAGC	CCTCACTAATAATTGAAACACG
				SSR (GTGTGC)6	*sun592*	TCTGGTGATTTTTGTTAGGAA	AACTCTGTGCATGCTAGTTTC
				SSR (CTGGAA)6	*sun593*	TCCTCCATCCTCTCTTCC	TTTATGGGTACGTGCTGTATT
1375819	5B_6422050	339,989,574	339,989,506	SSR (AG)6	*sun594*	TTGACTCTCTACCATCCAGAA	AACATATGACTGAAGGACCAA
				SSR (CGG)4	*sun595*	GTACAGCGAGACATGTGAGAC	GATGTGGAGAACCAAGTCAT

^a^ M13 sequence: CACGACGTTGTAAAACGAC was added at the 5′ end.

**Table 2:** Kompetitive Allele-Specific polymerase chain reaction (KASP) markers, designed for single-nucleotide polymorphisms (SNPs) and detected in sequenced parental sequence-tagged site (STS) amplicons.

STS Marker	KASP Marker	Allele 1 Primer ^a^	Allele 2 Primer ^b^	Common Primer
*sun849*	*sunKASP_238*	GGCTGTCCGCAAATCTTC	GGGCTGTCCGCAAATCTTA	GACAAGTCCGAACATCCACA
	*sunKASP_239*	GGCTATGGCTGATGGAAGAA	GGCTATGGCTGATGGAAGAG	CGACCACAACTCAGCAAAGA
	*sunKASP_240*	TGGATCCACCGAGAAGGA	GGATCCACCGAGAAGGG	CCCTCCTTCCTTCCTTCCTT
	*sunKASP_241*	ACATGCACGTCCACAAACA	CACATGCACGTCCACAAACT	CGTTGTGTGCCAGCTAGAAG
	*sunKASP_242*	TAACTCCAACGGACCCTCAA	TAACTCCAACGGACCCTCAC	GACGATGTGTAGCTCTTGTGG
	*sunKASP_243*	CTCTCCCTCATTCGCTCAG	CTCTCCCTCATTCGCTCAC	TGTCCGGTGTGCTCCTTATT

^a^ A1 primer labeled with FAM: GAAGGTGACCAAGTTCATGCT; ^b^ A2 primer labeled with HEX: GAAGGTCGGAGTCAACGGATT.

**Table 3:** Genotyping of diverse wheat germplasm using *Sr48*-linked marker *sunKASP*_239.

Cultivars	Marker Allele
Arina	A:A
Cezanne	*G:G*
*Australian cultivars*	
AGT Katana, Axe, Baxter, Beaufort, Bolac, Calingiri, Carnamah, Catalina, Chara, Chief CL Plus, Cobra, Coolah, Corack, Correll, Crusader, Dart, Derrimut, DS Faraday, EGA Bonnie Rock, EGA Bounty, EGA Burke, EGA Gregory, EGA Wedgetail, EGA Wylie, Elmore CL PLus, Emu Rock, Envoy, Espada, Estoc, Forrest, Fortune, Gauntlet, Gazelle, GBA Sapphire, Giles, Gladius, Grenade CL Plus, Harper, Impala, Impose CL Plus, Janz, Justica CL Plus, King Rock, Kord CL Plus, Kunjin, Lancer, Lang, Lincoln, Livingston, LRPB Arrow, LRPB Flanker, LRPB Kittyhawk, LRPB Reliant, Mace, Mackellar, Magenta, Mansfield, Merinda, Merlin, Ninja, Orion, Phantom, Preston, Scout, Sentinel, SF Adagio, SF Scenario, Shield, Spitfire, SQP Revenue, Strzelecki, Sunco, Sunguard, Sunmax, Suntop, Sunvale, Sunzell, Trojan, Ventura, Waagan, Wallup, Wedin, Westonia, Wyalkatchem, Wylah, Yandanooka, Yitpi, Young	G:G
Naparoo	A:A
*European cultivars*	
Apu, Atson, Bastian, Batalj, Bjarne, Børsum, Brons, Canon, Dala, Dalarna, Diamant, Diamant ll, Drabant, Dragon, ELS 6404-102-3, Extra Kolben, Fagott, Fram l, Fram ll, Fylgia l, Fylgia ll, Haarajärvi ME0102 Apu, Halland, Horsmanaho ME201 Timantti, J-03, Järvenkylä ME0302 Timantti, JO 3524, JO 8023, Jokikylä ME0505 Apu, Kärn, Kärn ll, Kimmo, Kiuru, Laitiala AP0103, Landvårkveite, Lantvete från Dalarna, Lantvete från Halland, Lavett, Manu, Monola ME1301, Møystad, MS 273-150, Naxos, Nemares, Nora, Norrøna, østby, Polkka, Pompe, Pondus, Prins, Progress, Rang, Reno, Ring, Rival, Rollo, Rubin, Runar, Ruso, Saffran, Safir, Sappo, Sibirian, Skirne, Snøgg II, Snøgg l, Sober, Sopu, Sport, Svenno, Timantti, Timantti Paavo, Tjalve, Touko, Troll, Trym, Ulla, Vinjett, Walter, Zebra	G:G
Avle, Blanka, Boru, Dacke, Kadett, Vitus, William, WW 20299	A:A

## Data Availability

All data are provided within the manuscript. Publicly available statistical tools were used in this study.

## Supplementary Materials

The following supporting information can be downloaded at: https://www.mdpi.com/article/10.3390/plants12081601/s1, Table S1: Distribution of markers in the different linkage groups of Arina/Cezanne RIL population.
