# Supplementary material for: Relocation of Sr48 to Chromosome 2D Using an Alternative Mapping Population and Development of a Closely Linked Marker Using Diverse Molecular Technologies
Source: Plants (Basel). 2023 Apr 10;12(8):1601. doi: 10.3390/plants12081601 (PMC10142899; doi:10.3390/plants12081601)
Supplement: Supplementary file 1 [file plants-12-01601-s001.zip › plants-2262432-supplementary.pdf]

Supplementary Table S1 Distribution of markers in the different linkage groups of Arina/Cezanne RIL population.

Chromosome	Markers per chromosome	Chromosome length (cM)
A genome		
1A.1	4	7.4
1A.2	17	15.8
1A.3	34	13.1
2A	493	283.7
3A.1	49	10.7
3A.2	302	236.3
4A	393	188.9
5A.1	257	140.3
5A.2	64	57.8
6A.1	114	58.7
6A.2	50	63.4
6A.3	64	67.9
7A.1	379	166.1
7A.2	149	119.0
**Total**	**2369**	**1429.0**
B Genome		
1B.1	513	142.2
1B.2	86	48.0
2B.1	83	24.3
2B.2	703	209.9
3B.1	424	142.1
3B.2	58	16.2
3B.3	57	19.5
3B.4	2	0.5
4B.1	55	15.1
4B.2	200	127.4
5B.1	65	35.8
5B.2	74	32.1
5B.3	195	98.2
6B	690	222.9
7B.1	118	70.8
7B.2	14	11.3
7B.3	276	82.3
**Total**	**3613**	**1298.6**
D Genome		
1D.1	17	7.9
1D.2	42	27.8

1D.3	46	75.7
1D.4	8	12.0
2D.1	239	134.2
2D.2	66	63.6
3D.1	77	21.3
3D.2	35	23.0
3D.3	18	28.9
3D.4	39	36.2
4D.1	4	1.1
4D.2	36	80.2
5D.1	66	39.1
5D.2	13	20.4
5D.3	28	54.7
5D.4	69	31.3
6D.1	7	7.5
6D.2	59	64.2
6D.3	81	26.3
6D.4	3	4.1
7D.1	71	33.5
7D.2	93	97.2
7D.3	16	35.6
**Total**	**1133**	**925.9**
**Total of A+B+ D Genome**	**7115**	**3653.5**
